# From Pathophysiology to Surgical Treatment: A Comprehensive Narrative Review of Ketamine-Induced Cystitis

**DOI:** 10.7759/cureus.117970

**Published:** 2026-10-15

**Authors:** Kisshan Raj Balakrishnan, Cassandra Ramadass, Kasthuri Nallathamby, Shaarven Kumar, Nityanandan G

**Affiliations:** 1 Urology, Wrexham Maelor Hospital, Wales, GBR; 2 Care of the Elderly, Glan Clwyd Hospital, Wales, GBR; 3 Clinical Oncology, The Christie NHS Foundation Trust, Manchester, GBR; 4 Clinical Oncology, Weston Park Teaching Hospital, Sheffield, GBR

**Keywords:** augmentation cystoplasty, bladder pain, interstitial cystitis, ketamine-induced cystitis, ketamine uropathy, lower urinary tract symptoms, recreational drug misuse

## Abstract

Ketamine-induced cystitis (KIC) is an increasingly recognised complication of recreational ketamine use, predominantly affecting young adults and causing severe lower urinary tract symptoms, bladder pain, and progressive structural damage. Its pathophysiology involves urothelial toxicity, chronic inflammation, neurogenic sensitisation, and progressive fibrosis, with advanced disease potentially resulting in a contracted, poorly compliant bladder and upper urinary tract compromise. This narrative review summarises current evidence on the epidemiology, pathophysiology, clinical presentation, diagnosis, and management of KIC, including its distinction from interstitial cystitis/bladder pain syndrome. A literature search was conducted in November 2025 using MEDLINE, Embase, PubMed, and Google Scholar, with relevant literature identified using combinations of terms including "ketamine", "ketamine-induced cystitis", "ketamine cystitis", and "ketamine uropathy". Ketamine cessation remains the cornerstone of management and may improve symptoms and early inflammatory changes. Medical and intravesical therapies may provide symptomatic benefit, although the supporting evidence is predominantly derived from observational studies and small case series. Patients with advanced bladder contracture, poor compliance, or upper urinary tract compromise may require reconstructive surgery or urinary diversion. Early recognition, ketamine cessation, multidisciplinary addiction support, and appropriate escalation of urological management are central to limiting irreversible urinary tract damage.

## Introduction and background

Ketamine is a dissociative anaesthetic that acts predominantly as a non-competitive N-methyl-D-aspartate (NMDA) receptor antagonist. Since its introduction in the 1960s, it has been widely used for anaesthesia, procedural sedation and analgesia, with more recent applications in treatment-resistant depression. However, recreational ketamine use has increased substantially, particularly among adolescents and young adults, raising concerns regarding its long-term systemic complications [1-3].

Ketamine-induced cystitis (KIC) is a recognised complication of chronic ketamine exposure and was first described in patients presenting with severe lower urinary tract symptoms (LUTS) and bladder pain associated with recreational use [4,5]. Subsequent reports from several countries have established KIC as a potentially progressive urological disorder characterised by urinary frequency, urgency, dysuria, haematuria, and pelvic pain, often accompanied by reduced bladder capacity and compliance. Continued ketamine exposure may result in progressive bladder fibrosis, ureteric involvement, hydronephrosis, and, in severe cases, renal impairment [4-7].

Early recognition remains challenging because patients may not disclose recreational ketamine use due to stigma or concerns regarding legal consequences, while the clinical presentation may overlap with other causes of chronic bladder pain and LUTS. Management is further complicated by variation in disease severity and the coexistence of ketamine dependence. Ketamine cessation remains central to treatment, while additional medical, intravesical or surgical interventions may be required according to disease severity [4-7].

This narrative review aims to critically summarise current evidence on the epidemiology, pathophysiology, clinical presentation, diagnostic evaluation and management of KIC, with particular emphasis on distinguishing KIC from interstitial cystitis/bladder pain syndrome (IC/BPS), evaluating the strength and limitations of available therapeutic evidence, and outlining the role of multidisciplinary and surgical management in advanced disease.

Methods

A narrative literature search was conducted in November 2025 using PubMed, MEDLINE, Embase, and Google Scholar. Search terms included combinations of "ketamine", "ketamine-induced cystitis", "ketamine cystitis", and "ketamine uropathy." The search aimed to identify literature relevant to the epidemiology, pathophysiology, clinical presentation, diagnostic evaluation, differential diagnosis, and management of KIC. Reference lists of relevant publications were also reviewed to identify additional pertinent studies.

Articles were selected according to their relevance to the objectives of this narrative review. Original clinical studies, observational studies, case series, reviews, and relevant consensus or guideline publications were considered. Contemporary evidence was prioritised where available, while earlier seminal studies were retained where they provided important historical, clinical, or mechanistic information. Experimental and translational studies were also considered where relevant to the proposed pathophysiological mechanisms of KIC, with these findings distinguished from clinical evidence during interpretation.

The identified literature was synthesised narratively. Where multiple publications addressed the same topic, greater emphasis was placed on more recent evidence, larger clinical studies, systematic reviews, and consensus or guideline publications where available. As this was a narrative review rather than a systematic review, the search was intended to identify clinically relevant and representative literature rather than to provide an exhaustive systematic retrieval of all published studies. Accordingly, no formal PRISMA-based study-selection process, meta-analysis, or structured risk-of-bias assessment was undertaken.

Epidemiology

The data on epidemiology have surged considerably since the initial coining of KIC. However, it is vital to note that precise estimates remain limited due to the illicit nature of ketamine misuse and the apprehension of individuals affected, which limits their ability to pursue and remain enlisted in care. The distribution of KIC globally is strongly mirrored by recreational ketamine use. Southeast and East Asia are reported to have the highest disease burden. Countries such as Hong Kong, Taiwan, Malaysia, and Singapore were among the pioneers in documenting cases involving large clusters, again mirroring the high prevalence of ketamine misuse in the youth and club subcultures [5-7]. Focusing on Hong Kong, where the most abused psychotropic substance among young adults in the mid-2000s was ketamine, observational studies conducted then reported that between 30% and 50% of frequent users developed urinary tract symptoms consistent with KIC [8]. On the other hand, a nationwide cross-sectional study in Taiwan depicted a similarly high prevalence of urinary tract symptoms in the midst of chronic ketamine users, with their severity in direct correlation with the cumulative exposure duration to ketamine. Studies conducted in hospitals in Taipei and Kaohsiung further reported that 20% of individuals who exhibited urological symptoms then illustrated radiological evidence of hydronephrosis or ureteric stricture at the time of presentation, outlining the aggressive nature of KIC in chronic users [9]. In the United Kingdom, a sharp increase was noted in ketamine misuse following 2010, highlighting the people in the age group of 16-24 years. Rising presentations of ketamine-related detriments were noted in the national data alongside urological case series describing increased KIC referrals, with a vast majority requiring surgical intervention. A retrospective cohort study conducted in London signified a threefold increase in the diagnosis of KIC between the years 2015-2021, which was in accordance with the rising availability of ketamine in the streets and its prevalence in festival and nightlife settings [4-7]. This similar epidemiological pattern was shared in another Western Country, Canada, with its emergency departments reporting a substantial increase in ketamine use alongside urological complications, specifically in metropolitan regions with vibrant nightlife. North American counterparts remain less well characterised than in Asia, but available evidence again shows a rising trend [4-7,10]. Across all regions, certain conclusions could be drawn. Affected individuals typically tend to fall within the 15-35-year age group, and a vast majority report daily or near-daily ketamine use for a period of 6-12 months before exhibiting urological symptoms. Male predominance was noted in previous studies, but this was quickly debunked by more recent studies showing a roughly equal sex distribution, which signifies that the risk is primarily due to exposure rather than biological vulnerability.

Several risk factors have been proposed as causing the development of KIC. The strongest predictors appear to be the frequency and duration of ketamine use. Consumption of ketamine >3-4 times per week or daily usage is associated with significantly increased risk. Correlation was also drawn between cumulative lifetime exposure and disease severity, with a number of studies identifying a dose-response relationship between average grams consumed in a month and the extent of bladder fibrosis. Another risk factor outlined was poly-drug use. Concomitant consumption of cocaine, MDMA, or amphetamines is shown to exacerbate urinary symptoms via a synergistic neurotoxicity mechanism or dehydration, though evidence remains mixed. A well-documented risk factor seems to be delayed presentation due to fear of legal consequences, stigma, and a lack of awareness. This subgroup, who happen to be the majority, usually present with advanced disease and often with bladder capacities of < 150 mL alongside upper tract involvement on first presentation [11,12]. Despite increasing data being presented in this field, epidemiological understanding remains incomplete. Several countries have conducted systematic surveillance studies on ketamine use and even less-tracked urological complications. Moreover, patients tend to reduce or cease the use of ketamine prior to seeking care, which further contributes to the discrepancy in the data collected in relation to true incidence. Hence, the dynamism of the epidemiological profile of KIC needs to be appreciated and is likely to evolve with the shift in drug trends, purity, and patterns of recreational use.

The available epidemiological evidence should be interpreted with caution. Much of the literature is derived from observational studies, retrospective cohorts, surveys, and specialist-centre case series, with substantial heterogeneity in the definition of ketamine exposure and KIC. Estimates of prevalence and disease severity may therefore be influenced by selection bias, under-reporting of recreational drug use, and differences in patterns of ketamine exposure between populations. Furthermore, a considerable proportion of the published evidence originates from regions with historically high ketamine use, particularly East and Southeast Asia, which may limit generalisability to other populations. Although an association between greater ketamine exposure and more severe urinary tract symptoms has been reported, the predominantly observational nature of the evidence limits causal inference and precise estimation of risk.

## Review

Pathophysiology

The pathophysiology of KIC is multifactorial and involves urothelial injury, chronic inflammation, neurogenic sensitisation, and progressive fibrotic remodelling. Current understanding is derived from a combination of human histopathological studies, animal models, and in vitro experiments; therefore, the relative contribution of individual mechanisms in humans remains incompletely defined [13-15].

Urothelial Injury and Inflammation

Ketamine and its metabolites, including norketamine, are excreted in the urine and have been implicated in direct urothelial injury. Experimental studies demonstrate disruption of epithelial integrity and altered expression of tight-junction proteins, including zonula occludens-1 (ZO-1) and E-cadherin, resulting in increased urothelial permeability [16]. Chronic ketamine exposure has also been associated with urothelial denudation and impaired epithelial regeneration in experimental models, while disruption of the glycosaminoglycan (GAG) barrier may further increase exposure of the underlying bladder wall and sensory nerves to urinary irritants [16-18].

Histological specimens from patients with KIC demonstrate inflammatory cell infiltration, including mast cells, eosinophils, lymphocytes, and macrophages. Increased expression of inflammatory and profibrotic mediators, including tumour necrosis factor-alpha (TNF-α), interleukin-6 (IL-6), and transforming growth factor-beta (TGF-β), has also been described [19,20]. TGF-β may contribute to the transition from persistent inflammation to fibrosis through fibroblast activation and collagen deposition. Similar inflammatory changes have been demonstrated in experimental models, although translation of these findings to human disease remains an area of ongoing investigation [19,20].

Neurogenic Sensitisation

Neurogenic mechanisms may contribute to the severe pain, urgency, and urinary frequency associated with KIC. Altered expression of sensory receptors, including transient receptor potential vanilloid-1 (TRPV1), has been demonstrated in bladder tissue from patients with KIC and may contribute to increased afferent sensitivity [20-26]. Peripheral and central sensitisation have also been proposed as contributors to persistent bladder pain; however, much of the mechanistic evidence in this area remains derived from experimental and translational studies rather than large clinical cohorts [26,27].

Fibrotic Remodelling and Upper Urinary Tract Involvement

Persistent inflammation may lead to progressive fibrotic remodelling, with collagen deposition, smooth muscle disruption, reduced bladder capacity, and impaired compliance [28,29]. In advanced disease, this may result in a severely contracted bladder with high storage pressures, potentially contributing to vesicoureteral reflux, hydronephrosis, and deterioration of renal function [26-29].

Established fibrotic remodelling may persist despite ketamine cessation, particularly in advanced disease. Patients with severe, refractory bladder contracture and poor compliance may require reconstructive surgery, such as augmentation cystoplasty [18].

Upper urinary tract involvement has been reported in severe KIC and may include hydronephrosis, ureteric obstruction or strictures, and renal impairment [18,26-29]. Proposed mechanisms include transmission of elevated intravesical pressure to the upper urinary tract and direct or peri-ureteric inflammatory and fibrotic changes. However, estimates of the frequency of upper tract involvement vary between studies, reflecting differences in patient populations, disease severity, and study design.

Overall, current evidence supports a multifactorial model in which urothelial injury and barrier dysfunction promote inflammation and sensory nerve activation, followed by progressive fibrotic remodelling in more advanced disease. However, much of the mechanistic evidence is derived from observational studies and experimental models, and further clinical research is required to clarify the relative importance and reversibility of these pathways in humans.

Clinical features

KIC typically presents with severe lower urinary tract symptoms (LUTS) and bladder pain, most commonly in young adults with a history of regular ketamine use. Common symptoms include urinary frequency, urgency, dysuria, nocturia, suprapubic pain, and haematuria [29-31]. The severity of symptoms varies considerably according to the extent of urinary tract involvement and continued ketamine exposure.

Urinary frequency can be particularly severe, with some affected patients reporting very frequent voiding during both the day and night [29-31]. This is associated with progressive reduction in functional bladder capacity and compliance. Urgency and dysuria are also frequently reported. Although urothelial inflammation, barrier dysfunction, and sensory nerve sensitisation have been proposed as mechanisms contributing to these symptoms, much of the mechanistic evidence is derived from observational and experimental studies rather than prospective clinical studies [29-31].

Bladder and suprapubic pain are prominent features of KIC and may worsen with bladder filling and improve temporarily following voiding. In more advanced disease, pain may become persistent and difficult to manage [32-35]. Haematuria may be microscopic or macroscopic and has also been reported in patients with KIC, particularly in the presence of significant mucosal inflammation or ulceration [10,36]. However, reported frequencies of individual symptoms vary between studies, reflecting differences in study populations, definitions of KIC, and severity of ketamine exposure.

Progressive reduction in bladder capacity is an important feature of advanced KIC. Cystoscopic and urodynamic studies have demonstrated markedly reduced bladder capacities and impaired compliance in severely affected patients [29-37]. Continued ketamine exposure has been associated with more severe urinary symptoms and structural bladder abnormalities, although the available evidence is predominantly observational and does not establish a uniform rate of disease progression [29-37].

KIC can also have substantial effects on quality of life. Severe urinary frequency, nocturia, chronic pain, and sleep disruption may impair occupational, academic, psychological, and social functioning [29-35]. Sexual symptoms, including dyspareunia and painful ejaculation, have also been described, although these outcomes are less consistently evaluated in the available literature [33-35]. Psychological distress and ketamine dependence may coexist and can further complicate treatment and cessation.

In advanced disease, upper urinary tract involvement may occur, including ureteric obstruction or strictures, hydronephrosis, and renal impairment [29-37]. Patients may consequently present with flank pain, recurrent urinary tract infection, or deterioration in renal function. These complications emphasise the importance of assessing the upper urinary tract in patients with severe or progressive disease.

The natural history of KIC remains incompletely characterised. Available observational evidence suggests that continued ketamine exposure is associated with persistent or progressive symptoms, whereas cessation may result in symptomatic improvement, particularly in earlier disease [29-37]. Established structural abnormalities and fibrosis may be less reversible. Nevertheless, the extent and rate of recovery vary between patients, and current evidence does not support a uniform trajectory for all individuals with KIC.

Diagnostic evaluation

A systemic, multimodal approach involving the integration of presenting clinical history, laboratory testing, imaging, cystoscopy, and urodynamic assessment is needed for the diagnostic evaluation of KIC. Due to the overlapping nature of symptoms with various inflammatory and non-inflammatory bladder disorders, and due to the underreporting of ketamine use, the early identification and diagnosis of KIC is deemed challenging. As a consequence of this, KIC is often misdiagnosed initially and misattributed to recurrent urinary tract infection (UTI), sexually transmitted infections, overactive bladder (OAB), or IC/BPS. This necessitates a structured diagnostic algorithm to ensure timely diagnosis and to curb irreversible bladder fibrosis, upper urinary tract pathology, and renal impairment [34,35]. The cornerstone of diagnosis remains a well-taken, detailed clinical history. Clinicians should emphasise the onset of symptoms, duration, and the severity of LUTS, as well as corresponding suprapubic pain, nocturia, haematuria, and sexual symptoms. Emphasis should also be placed on the exploration of ketamine use. Frequency, duration, route, and amount of use are useful in helping the patient; however, a vital challenge here would be that patients are usually not forthcoming due to stigma and apprehension of legal repercussions. This is evidenced in studies where up to 40% of patients are noted to have denied the use of recreational drugs, and full disclosure is only achieved after mutual trust and rapport are established [36,37]. A high index of suspicion should always be maintained when assessing young adults presenting with severe bladder symptoms unexplained by infection or anatomical abnormalities, as ketamine users normally fall in the age range of 15-35 years old.

There are certain historical clues which point to a diagnosis of KIC, which are detailed in Table 1 [33-37].

**Table 1:** Main historical clues suggestive of ketamine-induced cystitis. Source: [33-37]

Main historical clues suggestive of ketamine-induced cystitis
Rapidly progressing LUTS over the period of weeks to months
Severe urinary frequency which is inordinate to voided volumes
Suprapubic pain where transient relieve is achieved post voiding
Recurrent ‘sterile UTIs’ which are culture negative
Ketamine binges leading to symptoms exarcebation

Laboratory Evaluation

Urinalysis is not a reliable diagnostic marker. Tsai et al. described that underlying epithelial inflammation in the absence of infection manifests as sterile pyuria, which is the most common finding [10]. Mak et al. further reported that microscopic haematuria is found in 20-50% of the cases, which explains mucosal ulceration and neurovascular fragility [9]. When present, proteinuria is usually mild and represents inflammatory exudation rather than glomerular pathology. Nitrites are usually lacking unless there is superimposed infection at the same time. Negative cultures are a reliable marker to differentiate KIC from bacterial cystitis. Nonetheless, recurrent empirical antibiotic use is observed before diagnosis. In a young patient, persistent LUTS with negative cultures prompts evaluation for KIC. To assess for upper urinary tract involvement, serum creatinine, urea, and estimated glomerular filtration rate (eGFR) should be measured to assess for upper urinary tract involvement. It needs to be mentioned, however, that renal function may be normal in the earlier stages. Hydronephrosis and obstructive uropathy can contribute to rising creatinine in advanced cases [36-38]. Li et al. reported that several small studies have identified elevated urinary cytokines such as IL-6, MCP-1, and NGF in KIC. These markers, however, remain investigational and are not routinely used in day-to-day clinical practice [8].

Imaging Studies

The first-line imaging modality is an ultrasound due to its accessibility and lack of exposure to radiation. Key findings that can be gathered include bladder wall thickening, reduced bladder capacity, trabeculation, perivesical hypervascularity, and hydronephrosis in advanced cases. Hydronephrosis is observed in approximately 10-30% of severe KIC cases and indicates high-pressure voiding or ureteric obstruction [36]. A more detailed and comprehensive evaluation is provided by CT urography and is recommended when severe disease or upper tract complications are suspected. Common findings are mural thickening of the bladder, ulceration or mucosal irregularity, perivesical fat stranding, ureteric narrowing or stricture, and hydronephrosis. Chan et al. reported that CT is especially useful in distinguishing KIC from malignancy, ureteric stones, and anatomical causes for obstruction [13]. MRI is not routinely required, but may well aid in situations where delineation of the soft tissue, pelvic floor involvement, and fibrosis of the bladder wall is required. However, it is important to note that there are limited studies to support the use of MRI to better differentiate chronic fibrosis from active inflammation. The decisive diagnostic tool in cases of KIC, which provides direct visual evidence of KIC-perpetrated bladder damage, is cystoscopy [33-36]. Characteristic evidence that could be gathered via direct visualisation of the bladder includes glomerulations, mucosal cracking or fissures, erythema and ulceration, exposed vasculature, reduced bladder capacity, and pseudomembranes in severe inflammation. A biopsy could also be obtained, and these would typically reveal chronic inflammatory infiltrates, urothelial denudation, lamina propria oedema, mast cell infiltration, and early fibrosis. Correlation can be drawn between the appearance of the bladder under cystoscopy and the severity of the disease and presenting clinical symptoms. Cystoscopy also serves as an important tool to differentiate KIC from IC/BPS, which more typically presents with Hunner lesions in a subset of patients. Urodynamic studies are noted to be pivotal to quantify the functional impact due to KIC and to guide the management plan. Common findings here include reduced bladder capacity, poor compliance, detrusor overactivity, and early sensory urgency. Li et al. have confirmed that bladder capacity is often markedly reduced, sometimes to less than 100 mL due to fibrosis of the bladder and limited filling of the bladder due to pain [8]. The compliance of the bladder is also severely reduced, causing steep pressure-volume curves and elevated intravesical pressures during the storage phase. This discovery robustly corresponds with the risk of upper tract deterioration. Chronic inflammation and neurogenic sensitisation lead to detrusor overactivity, which is evidenced by patients often reporting severe urgency during filling of the bladder. At bladder volumes as low as 10-20 mL, pain or pressure sensations may present in patients owing to urothelial denudation and afferent hyperexcitability. Hence, the confirmation from urodynamics of poor compliance and reduced bladder capacity is necessary to assess the need for surgical intervention, augmentation cystoplasty in particular [33,36].

Differential Diagnosis

Due to the nature of the symptoms that someone with KIC presents, the differential diagnosis is often broad and includes both infectious and non-infectious conditions [33-35]. Important conditions to distinguish include recurrent UTIs, with patients having positive cultures and systemic symptoms alongside them. Infective cystitis or bladder pain, on the other hand, typically presents with a slower progression of symptoms, and characteristic Hunner lesions are noted on direct visualisation of the bladder. Radiation cystitis is a differential in someone with a history of pelvic radiation, whereas radiation cyclophosphamide-induced cystitis is plausible in someone with chemotherapy exposure. TB cystitis should also be excluded in someone with sterile pyuria and caseating granulomas on histology. Other common conditions should also be excluded before reaching the diagnosis of KIC, including endometriosis, where a female patient could present with cyclical pain and urolithiasis, which warrants radiology scans to rule out stones. A combination of negative urine cultures, severe symptoms, rapid progression, and a young patient should raise alarm bells, and a clinician should have heightened suspicion for KIC.

Diagnostic Algorithm

In cases of KIC, a structured approach increases the accuracy of diagnosis. Table 2 showcases the proposed algorithm for diagnosis of KIC in accordance with the British Association of Urological Surgeons Consensus statements on the management of ketamine uropathy [32-36].

**Table 2:** Proposed diagnostic algorithm for ketamine-induced cystitis. Source: [32-36]

Proposed diagnostic algorithm for ketamine-induced cystitis	
1. Initial clinical assessment	Comprehensive urological history and examination
2. Baseline investigations	Urinalysis, urine culture and cytology, full blood count, urea and electrolytes, liver function tests
3. Symptom and quality-of-life assessment	Pelvic Pain and Urgency/Frequency (PUF) questionnaire; International Consultation on Incontinence Questionnaire-LUTS (ICIQ-LUTS)
4. Ketamine-specific history	Duration of ketamine use and route of administration; estimated dose or grams per week
5. Functional bladder assessment	Uroflowmetry; post-void residual measurement (PVR) to determine functional bladder capacity; three-day bladder diary
6. Endoscopic evaluation	Cystoscopy ± biopsies when clinically indicated
7. Renal and upper tract imaging	Renal ultrasonography
If hydronephrosis detected:	MAG3 renogram, CT urogram, video urodynamic studies (UDS)

The diagnosis of KIC requires a high index of clinical suspicion alongside appropriate multimodal investigation. Early recognition is important, as ketamine cessation offers the greatest opportunity for symptomatic and inflammatory improvement before advanced structural and fibrotic changes develop [18]. A structured diagnostic approach can help distinguish KIC from other bladder pathologies and guide subsequent management.

Comparison between KIC and IC/BPS

Several key clinical features are shared between KIC and IC/BPS. These include chronic pelvic pain, urinary frequency, and urgency. It is vital to note that these two conditions have distinct pathophysiology. Clinical differentiation is imperative, as KIC progresses more rapidly, causes greater structural damage, and generally warrants different management strategies that incorporate ketamine cessation, early consideration for surgery, and targeted interventions to curb addiction to the substance. The section below details the contrast in the epidemiology, pathophysiology, signs and symptoms, findings in cystoscopy, histopathology, urodynamics, and the treatment response [37,38]. In contrast to KIC, IC/BPS tend to affect mainly middle-aged women, with peak incidence noted between the ages of 40-60 and noted to be predominantly idiopathic. KIC tends to dominate in the 25-35 age group and shows a dose-response relationship with recreational ketamine use, as previously described. In comparison to IC/BPS, where the incidence is noted to be stable globally, KIC is dependent on ketamine misuse. Mak et al. have evidenced geographical clustering of KIC in regions with high recreational consumption of ketamine in countries such as Hong Kong, Taiwan, Malaysia, and the United Kingdom [9]. Sex distribution was also noted to differ. Tsai et al. discussed that IC/BPS was weighted more towards females in a 5.1 distribution, whereas KIC showed a balanced prevalence between sexes or a mildly male-predominant distribution. This again consolidates a recreational drug use pattern rather than biological susceptibility in KIC [10]. Both disorders have a similar pattern of disruption in regard to the bladder, where there is a disruption of the urothelial barrier; however, the mechanisms differ. IC/BPS is defined by thinning of the epithelia of the bladder, antiproliferative factor upregulation, and increased activity of the mast cell. Where both KIC and IC/BPS cause an increase in urothelial permeability, structural destruction is uncommon in IC/BPS. As discussed prior, KIC involves direct ketamine and its substrate norketamine-induced urothelial toxicity, tight-junction disruption, and broad epithelial denudation [39,40]. Each condition induces inflammation; however, chronic and low-grade inflammation is typically noted in IC/BPS, barring the subset with Hunner lesion, where intense inflammation and focal ulceration are noted. This, as previously discussed, is in contrast to KIC, where the inflammation is widespread, transmural, and marked by prominent eosinophils, mast cells, and lymphocyte infiltration [40,41]. Both conditions display upregulation of the sensory nerve and afferent hypersensitivity. However, a raised neurogenic sensitisation is demonstrated in KIC due to chronic exposure to ketamine use and dysregulation of the NMDA receptor [42].

A focal feature of KIC has been fibrosis of the bladder wall; however, this is noted to be uncommon in IC/BPS. Minimal fibrosis is noted on direct visualisation of the bladder in IC/BPS, except in conditions with Hunner lesions. This again is in contrast to KIC, where raised collagen deposition, smooth muscle destruction, and thickening of the bladder wall are noted, giving rise to a "thimble bladder" morphology. These characteristics demonstrate why KIC advances to end-stage bladder failure far more swiftly than IC/BPS [42,43]. A similar symptom spectrum is shared by both disorders, and it includes frequency, urgency, nocturia, and suprapubic pain. However, several distinguishable factors exist. KIC most notably progresses in a more aggressive pattern than IC/BPS, and Li et al. reported that most patients exhibit symptoms within months of daily ketamine use [8]. In IC/BPS, this typically progresses over the course of years. Furthermore, pain exhibited in KIC is typically more pronounced, intense, and persistent even when the bladder is not holding much volume, and rebound hyperalgesia causes immediate exacerbation after ketamine use. Pain fluctuation and characteristic relief are noted after voiding in IC/BPS. As for pain, frequency is also more pronounced in KIC, up to 40-60 voids per day, which reflects a bladder that is contracted and non-compliant. In IC/BPS, where frequency is still significant, higher voiding volumes are generally noted, and there is no structural fibrosis in the early days [38-43].

Clear distinctions can be made between KIC and IC/BPS via cystoscopy. A localised area of ulceration, preserved bladder capacity, and Hunner lesions in about 10-20% of the cases, alongside non-specific glomerulations after hydrodistension, are noted in IC/BPS. In KIC, on the other hand, widespread mucosal erythema, fissures and linear mucosal cracking, ulceration and pseudomembranous formation, fragile bleeding-prone mucosa, and markedly reduced bladder capacity are noted. Diffuse distribution of lesions is noted in KIC in combination with more profound epithelial destruction [38-42]. A key distinction is noted in the histology in these two conditions. Mild urothelial thinning, patchy lymphocytic infiltrate, increased mast cell density (especially perivascular), and (if present) focal fibrosis can be noted around Hunner lesions; while in KIC, as previously mentioned, urothelial denudation, ulceration with granulation tissue, dense chronic inflammatory infiltrate, widespread fibrosis in the lamina propria, smooth muscle degeneration, and neovascularisation are visualised. These findings again substantiate the argument that KIC is more catastrophic and a swiftly deteriorating condition [41,42]. A characteristically different pattern is noted in urodynamics in these two conditions. In IC/BPS, lowered bladder compliance is noted in some patients, whereas in the vast majority, they have a normal bladder capacity. They also exhibit pain at moderate filling volumes, and limited detrusor overactivity is noted. As discussed, a more distinct pattern is noted in KIC, where a severely reduced bladder capacity (< 100-150 mL), significant poor compliance, pain at significantly low bladder volumes (< 20 mL), and a high storage pressure leading to upstream pressure causing upper tract risk. These distinctions aid in determining prognostic outlooks and inform operative decisions [41,42].

Differentiating KIC from IC/BPS carries major therapeutic consequences. IC/BPS has shown improvement with behavioural therapy, pelvic-floor physiotherapy, and intravesical replenishment of the GAG layer. Oral antihistamines or neuromodulators and cystoscopic hydrodistension. In cases where symptoms are refractory to initial treatment, botulinum toxin or neuromodulation could be utilised. The emphasis is again on the fact that symptom progression is typically slow in IC/BPS, and invasive operation is seldom required. Similar treatment is refractory in KIC unless ketamine cessation has taken place. However, in KIC, patients would need intravesical therapy for symptomatic relief, high-dose neuropathic agents, botulinum toxin for urgency/pain, and, in advanced cases, reconstructive surgery for urinary diversion, including augmentation cystoplasty. Prolonged use of ketamine minimises the therapeutic impact of these interventions [41,42]. As discussed, prior, both KIC and IC/BPS have distinct investigation and management strategies. Where the progression in KIC is more rapid and necessitates early cessation of ketamine exposure alongside having a predisposition to causing hydronephrosis, strictures and renal impairment, IC/BPS seldom necessitates major reconstructive surgery. Additionally, patients in KIC require assistance in regard to addiction medicine, psychological support, and care from the multi-disciplinary team. Catastrophic bladder atrophy and preventable renal failure could be a result of misdiagnosing KIC as IC/BPS. In conclusion, albeit sharing overlapping presenting symptoms, substantial differences are noted in epidemiology, pathophysiology, structural implications, and treatment strategies. KIC is notably more aggressive, destructive, and rapid in progression, mandating early detection and proactive therapeutic action. Discriminating between these two conditions is vital to avert irreversible bladder fibrosis and protect upper urinary tract function [38-42].

Management of KIC

Management of KIC should be individualised according to symptom severity, ongoing ketamine exposure, bladder capacity and compliance, and the presence of upper urinary tract involvement. The principal aims are ketamine cessation, symptom control, preservation of bladder function, and prevention of renal impairment. Current treatment recommendations are based predominantly on observational studies, small case series, and expert consensus, with limited high-quality comparative evidence available to guide the optimal sequencing of therapies [33,44-50].

Ketamine Cessation and Multidisciplinary Management

Ketamine cessation is the cornerstone of management. Observational studies have consistently reported improvement in urinary symptoms following cessation, particularly in patients without advanced structural bladder damage [4,10,33]. Continued ketamine exposure is associated with persistent or progressive symptoms and may reduce the effectiveness of other treatments. However, the extent and timing of recovery following cessation vary considerably, and established fibrotic bladder contracture may persist despite abstinence.

Achieving sustained cessation can be challenging because of ketamine dependence and associated psychological or social factors. Management should therefore incorporate addiction services and psychological support where appropriate. Conservative measures, including adequate hydration and avoidance of recognised bladder irritants, may also be used for symptom management, although evidence supporting these interventions specifically in KIC is limited [33,49,50]. Pelvic-floor physiotherapy may be considered in selected patients with associated pelvic-floor dysfunction, but its use is largely extrapolated from experience in other chronic bladder pain conditions rather than KIC-specific studies.

Pharmacological and Intravesical Treatment

Pharmacological treatment is primarily directed towards symptom control and should be considered an adjunct to ketamine cessation rather than a substitute for it. Analgesics and neuropathic pain agents may be used for bladder and pelvic pain, while antimuscarinic or other bladder-directed therapies may be considered for storage symptoms. However, evidence for individual oral pharmacological treatments in KIC remains limited, and much of current practice is extrapolated from the management of other bladder pain and storage disorders [33,44-50].

Intravesical therapies have been investigated with the aim of restoring the urothelial GAG barrier and reducing inflammation. Hyaluronic acid and related GAG-replenishing treatments have demonstrated symptomatic and functional improvement in small observational studies and case series [47-49]. Nevertheless, the absence of large randomised controlled trials limits conclusions regarding their long-term efficacy and optimal treatment schedules.

Other interventions, including intravesical botulinum toxin A (BoNT-A), dimethyl sulfoxide (DMSO), and regenerative approaches such as platelet-rich plasma, have also been reported [33,44-50]. BoNT-A may improve urgency, frequency, and pain in selected patients, but evidence is largely based on small series, and benefit may be limited in patients with severely contracted, poorly compliant bladders. Evidence supporting DMSO and regenerative therapies in KIC remains particularly limited, and these approaches should therefore be regarded as adjunctive or emerging treatments rather than established standard therapy.

Management of Upper Urinary Tract Complications

Upper urinary tract involvement requires timely assessment because hydronephrosis, ureteric obstruction, and impaired renal function may occur in advanced disease. Management should address the underlying high-pressure or poorly compliant bladder where present, while ureteric stenting or percutaneous nephrostomy may be required for selected patients with significant obstruction or threatened renal function [51,52]. Continued monitoring of renal function and upper urinary tract imaging is appropriate in patients with severe disease.

Surgical Management

Reconstructive surgery is generally reserved for patients with advanced KIC who have severe bladder contracture, poor compliance, refractory symptoms, or upper urinary tract compromise despite ketamine cessation and appropriate conservative treatment [52-55]. Patient selection is particularly important, and sustained ketamine cessation should form part of the assessment for major reconstruction.

Augmentation enterocystoplasty is the most frequently reported reconstructive procedure for severe KIC. Published series have demonstrated improvements in bladder capacity, pain, urinary frequency, and functional outcomes following augmentation in appropriately selected patients [52-55]. However, these findings are derived predominantly from retrospective series with relatively small patient numbers and variable follow-up. Consequently, although reported outcomes are generally favourable, evidence does not permit definitive comparison with other reconstructive strategies.

Urinary diversion, with or without cystectomy, may be considered in selected patients with end-stage bladder dysfunction, severe refractory pain, recurrent infection, or when bladder preservation is not feasible [56-60]. The choice of reconstruction or diversion should be individualised according to bladder and upper tract function, patient preference, comorbidity, ability to undertake long-term follow-up, and the likelihood of sustained ketamine abstinence. Given the limited KIC-specific evidence comparing different reconstructive procedures, no single surgical approach can be considered universally superior.

Patients undergoing bowel-based urinary reconstruction require long-term follow-up because of potential renal, metabolic, infectious, and reconstructive complications. Ongoing addiction and psychological support also remain important following surgery, particularly because continued or recurrent ketamine exposure may compromise long-term outcomes [33].

Summary of Evidence

Ketamine cessation remains the foundation of KIC management, while conservative, pharmacological, and intravesical treatments may provide additional symptomatic benefit in selected patients. However, evidence for most non-surgical interventions is derived from observational studies and small case series, and robust comparative trials are lacking. Reconstructive surgery can provide substantial functional and symptomatic improvement in carefully selected patients with advanced disease, but the surgical literature is similarly dominated by retrospective series from specialist centres [33,47-60]. Treatment should therefore be individualised according to disease severity, bladder function, upper urinary tract involvement, response to cessation and conservative treatment, and patient factors.

For patients with KIC, a structured management pathway may facilitate appropriate escalation of treatment according to disease severity. Figure 1 summarises a proposed stepwise approach informed by available evidence and current consensus guidance [33,34,44-51,53-60].

**Figure 1:**
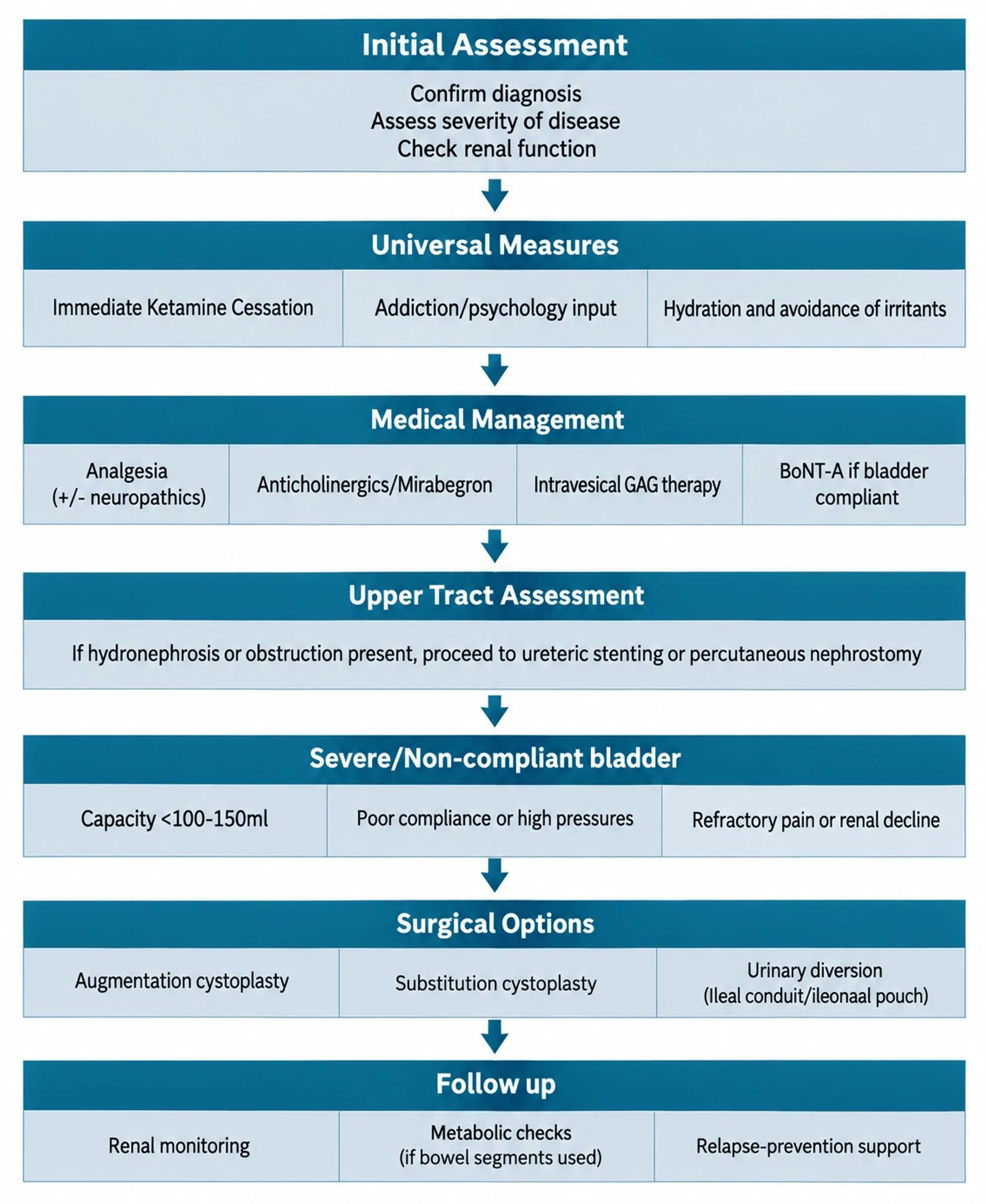
Proposed management algorithm for ketamine-induced cystitis. Source: [33,34,44-51,53-60]

Limitations

This review has several limitations. As a narrative review, it was not designed to provide an exhaustive systematic synthesis of the literature, and no formal PRISMA-based study-selection process or structured risk-of-bias assessment was undertaken. Furthermore, the available evidence on KIC is predominantly derived from retrospective observational studies, small case series, and specialist-centre populations, with considerable heterogeneity in definitions of ketamine exposure, disease severity, treatment response, and follow-up. These limitations restrict direct comparison between studies and mean that conclusions regarding treatment effectiveness and long-term outcomes should be interpreted cautiously.

## Conclusions

KIC is an increasingly recognised urological complication of ketamine use that can cause severe LUTS, bladder pain, and, in advanced cases, structural bladder and upper urinary tract abnormalities. Current evidence supports a multifactorial pathophysiology involving urothelial injury, inflammation, sensory dysfunction, and progressive fibrotic remodelling, although the relative contribution and reversibility of these mechanisms remain incompletely understood. Ketamine cessation remains the cornerstone of management and is associated with symptomatic improvement, particularly in earlier disease. Medical and intravesical treatments may provide additional symptomatic benefit, but the supporting evidence is predominantly derived from observational studies and small case series. Patients with advanced bladder contracture, poor compliance, or upper urinary tract compromise may require reconstructive surgery or urinary diversion. However, evidence comparing different treatment strategies remains limited.

Early recognition, support for sustained ketamine cessation, and multidisciplinary management involving urological, addiction, and psychological services are important components of care. Further prospective studies are required to better define the natural history of KIC, identify predictors of progression and recovery, and establish the comparative effectiveness and long-term outcomes of available treatments.
